# Cross-cultural similarity in relationship-specific social touching

**DOI:** 10.1098/rspb.2019.0467

**Published:** 2019-04-24

**Authors:** Juulia T. Suvilehto, Lauri Nummenmaa, Tokiko Harada, Robin I. M. Dunbar, Riitta Hari, Robert Turner, Norihiro Sadato, Ryo Kitada

**Affiliations:** 1 Department of Neuroscience and Biomedical Engineering, Aalto University, Espoo, Finland; 2 Department of Computer Science, Aalto University, Espoo, Finland; 3 Department of Art, Aalto University, Espoo, Finland; 4 Turku PET Centre, University of Turku, Turku, Finland; 5 Department of Psychology, University of Turku, Turku, Finland; 6 Institute of Biomedical and Health Sciences, Hiroshima University, Hiroshima, Japan; 7 Department of Experimental Psychology, University of Oxford, Oxford, UK; 8 Department of Neurophysics, Max Planck Institute for Human Cognitive and Brain Sciences, Leipzig, Germany; 9 National Institute for Physiological Sciences, Okazaki, Japan; 10 Division of Psychology, School of Social Sciences, Nanyang Technological University, 14 Nanyang Avenue, 637332 Singapore

**Keywords:** social touch, cultural differences, emotion, bonding

## Abstract

Many species use touching for reinforcing social structures, and particularly, non-human primates use social grooming for managing their social networks. However, it is still unclear how social touch contributes to the maintenance and reinforcement of human social networks. Human studies in Western cultures suggest that the body locations where touch is allowed are associated with the strength of the emotional bond between the person touched and the toucher. However, it is unknown to what extent this relationship is culturally universal and generalizes to non-Western cultures. Here, we compared relationship-specific, bodily touch allowance maps across one Western (*N* = 386, UK) and one East Asian (*N* = 255, Japan) country. In both cultures, the strength of the emotional bond was linearly associated with permissible touch area. However, Western participants experienced social touching as more pleasurable than Asian participants. These results indicate a similarity of emotional bonding via social touch between East Asian and Western cultures.

## 1. Introduction

Interpersonal touch is a critical part of human social communication. It contributes to cognitive and socioemotional development in childhood [1,2] and promotes relational, psychological and physical well-being in adulthood [3,4]. Given its importance, there has been growing interest in the effects of interpersonal touch on human social behaviour and in the resulting social relationships. Non-human primates spend remarkable amounts of time in grooming others, well beyond the necessity of removing parasites or vegetation debris from the fur [5]. Social grooming thus plays a particularly important role in social bonding, and the psychological experience of increased social closeness is reflected in prosocial behaviours [5,6]. In individual female primates, the social grooming patterns are explained by factors such as attraction to high-ranking individuals, attraction to kin and competition for grooming partners [7,8], implying that variations in the relationship specificity of social touch might be correlated with differences in social structure.

In our previous study, we asked 1368 people from Western countries (Finland, France, Italy, Russia and the UK) to indicate where on their body they would allow relatives, friends and strangers to touch them [9]. We also measured the emotional bond between them since such bonds are the best predictor for engaging in social contact with someone and as they track the position of different individuals in one's social network [10–12]. In each country, the topographic map of body areas that one is allowed to touch was associated with the strength of the emotional bond between the participant and the toucher. Thus, the relationship-specific patterns of social touch seem to be related to the establishment and maintenance of social structures and affective relationships among human adults.

However, these results cannot resolve whether bonding by social touch is a culturally universal phenomenon or specific to Western Europe which our previous study was limited to. Cross-cultural studies have shown that deeply embedded differences between Western and Eastern cultures strongly influence emotional processing, together with ideas regarding experiences such as emotions [13,14], the facially expressed emotions of others [15–17] and their integration with emotional voices [18]. Indeed, social touch varies according to culture [19–21]. For instance, North American students have more frequent physical contact than Japanese students with their friends and their parents [19]. Moreover, the US students' social touch patterns in regard to their fathers and mothers were similar, whereas Japanese students were physically closer to their mothers than their fathers. However, this study did not examine the relationship between the touching pattern and the strength of the emotional bonding. Thus, it remains unclear to which extent this relationship is culturally universal and generalizes to non-Western cultures.

Here we compared relationship-specific social touching patterns between one East Asian and one European culture. We used a high-resolution self-reporting tool (emBODY) to quantify relationship-specific maps of bodily regions where social touch is allowed. Participants in Japan and UK evaluated their emotional bonds with and drew touchable body regions for different social network members. We hypothesized that patterns of allowed interpersonal touch differ in Japan and in the UK but vary in a similar way as a function of social bond in both countries.

## 2. Material and methods

### (a) Participants

Altogether, 309 Japanese individuals and 622 British individuals participated in the study. The British sample was collected through Maximiles (data reported in [9]) and the Japanese sample was collected via MyVoice Communications, Inc., in 2016. As the population-level effect size was unknown, we aimed to have the sample in line with the first study reporting topographies of acceptable touch (altogether 1368 participants, average 274 per culture, [9])*,* which in turn were targeted to double the sample sizes from the earliest studies using this technology (total of 773 participants over seven separate experiments, [22])*.* After cultural background had been validated and quality control established (described in detail in the ‘Data analysis’ section), the data of 255 Japanese individuals (124 male, 40.1 ± 14.6 years) and 386 British individuals (214 male, 46.0 ± 12.6. years) were analysed. The British sample was chosen for comparison [9], (1) because it was collected via a paid service similar to the Japanese sample and (2) because the age distributions were similar in both groups.

### (b) Data acquisition

The data were collected online. Before beginning the study, participants gave informed consent online. The Japanese study protocol was approved by the ethics committee at the National Institute for Physiological Sciences, Japan. The English version of the online rating tool (emBODY, [9,22]; https://version.aalto.fi/gitlab/eglerean/embody) was first translated into Japanese by a professional translator (ZENIS Co., Ltd, Tokyo Japan). Then two researchers (R.K. and T.H.) revised the translated materials by conducting back-translation and translation until both were satisfied with the result.

Participants first provided background information about themselves and members of their social network. They were given a list of categories of relationship which may be found in one's social network (e.g. ‘aunt’, ‘female friend’) and participants then indicated if they had one or more individuals from these categories in their own social network. If participants had multiple individuals in their social network fitting one category, they were instructed to pick one individual and to answer all the subsequent questions regarding this individual. We also added ‘female stranger’ and ‘male stranger’ to the list of social categories, to probe the acceptable social touch with strangers.

Participants were then asked to give information about the sex (only for spouses) and ages of the selected members of their social network, as well as estimates of how long ago they last met them. We assumed that participants would encounter strangers on a daily basis and set time since the last meeting to 0 days for the strangers. We used participants' own age for the age of the strangers, and in subsequent tasks asked partners to respond with respect to ‘a woman/man of your age whom you do not know’. Next, the participants reported their emotional bond with each network member (scale from 1 representing no emotional bond to 10 representing the strongest possible emotional bond) and gave an estimate of how pleasant they would find being touched by each member of their social network (scale from 1, not pleasant at all, to 10, extremely pleasant).

After completing background questions, the participants were presented with the colouring task with the emBODY tool. They were asked to consider where on their bodies they would find it acceptable for different social network members to touch them in everyday situations. For each member of their social network, the participants were shown a body outline in both front and back view (electronic supplementary material, figure S1a) and asked to colour the bodily regions where each social network member would be allowed to touch them. The participants coloured the bodies using a computer mouse or, if they were completing the task using a mobile device, using their finger. Painting was additive so that multiple strokes on the same region increased opacity; this information was, however, not considered in the analyses (see below).

### (c) Data analysis

#### (i) Data preprocessing

The data were first checked for completeness. Data from the colouring tasks were then converted to Matlab (R2015b) two-dimensional matrices, where each cell represented a pixel on the screen. The size of the figures was 522 × 342 pixels (for front and back combined), out of which 89 129 pixels fell within the body outlines. The diameter of the painting tool was set to 17 pixels. The coloured images were binarized so that the amount of time a participant spent on colouring an area would not impact the results. Each participant completed between two and 15 individual touch area maps (TAMs), depending on the size of their social network.

As the aim of the study was to compare data between different cultures, cultural background criteria were used to exclude some participants. Japanese participants were asked about their parents' ethnicity, and whether they had spent an extended period of time abroad. We excluded any participants who did not report both of their parents’ ethnicity as ‘Japanese’ or who reported having spent more than a year abroad. Altogether, 17 Japanese participants were excluded based on the background questions. In the English version, the sample consisted of only British nationals. The participants were asked about their native (first) language and their cultural identity. We excluded all participants who did not report their first language as being English, or who did not identify themselves as ‘British’, ‘English’, ‘Irish’, ‘Welsh’ or ‘Scottish’. Altogether, 159 British participants were excluded based on the background questions. Finally, we visually inspected the TAMs and excluded 38 participants from Japanese dataset and 77 participants from British dataset due to inappropriate drawing (e.g. doodling or excessive colouring). The remaining samples, consisting of 386 British and 255 Japanese participants, were used for analysis.

#### (ii) Comparing the samples using two-proportion z-test

We compared the acceptable touch areas of the two cultural samples by comparing each pixel in each image using a two-proportion *z*-test, with a two-tailed alternative hypothesis, with *α* = 0.05, corrected for false detection rate with no correlation assumptions. To test the association between emotional bond and touchable body area, we first calculated a ‘touchability index’ (TI) as the proportion of coloured pixels within the body outline for each TAM [9]. To better quantify the differences in the topographies of acceptable touch, we then defined 13 anatomical regions of interest (ROIs) and calculated ROI-specific TIs as the proportion of coloured pixels within the ROI. We conducted multiple linear regression analysis on these TIs for each social network member in both countries, with the emotional bond and cultural background as explanatory variables. This analysis was conducted using group mean data and individual data.

## 3. Results

### (a) Touch area maps for Japanese and British individuals

**Figure 1.**
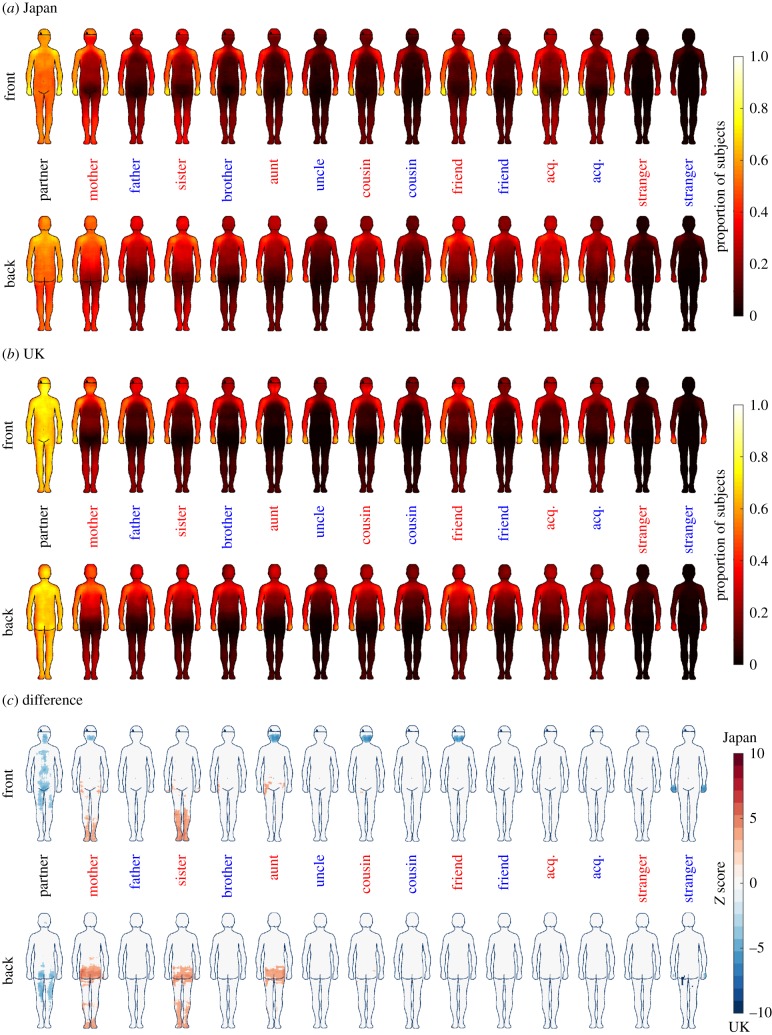
Relationship-specific TAMs in (*a*) Japan and (*b*) the UK. The colouring displays the proportion of the sample reporting that being touched by this person in this area is acceptable to them. (*c*) Comparison of the proportion of participants per culture who allow touching in different areas. Red colour in the maps indicates that Japanese participants reported that area more acceptable, blue colour indicates that British participants reported that area more acceptable. The data in (*c*) are thresholded at *p* < 0.05, FDR corrected. After FDR correction, *Z* threshold with no correlation assumptions varied from 3.22 to 5.98, depending on the number of participants who had that particular individual in their social network. Red and blue labels indicate female and male members of the social network, respectively.

Figure 1 shows the mean TAMs for different social members in Japanese and British samples.

The relationship-specific TAMs in the Japanese and UK populations were generally consistent. Specifically, the partner was allowed to touch basically anywhere on the body, and closest acquaintances and relatives were allowed to touch over the head and upper torso. By contrast, strangers were restricted to touch only the hands. Direct comparison of TAMs from British and Japanese participants by two-proportion *z*-test revealed that Japanese allowed more touching from their female relatives than did British, especially in the lower extremities and the bottom (figure 1*c*). On the other hand, more British participants allowed their partners to touch their bodies on the torso, face and legs. Moreover, British participants allowed their mother, aunt, female cousin and female friend to touch their heads more and male strangers to touch their hands more.

#### (i) Emotional bond and pleasantness ratings

In both countries, an individual's emotional bond was the strongest with their partner, followed by their closest family members and relatives. By contrast, participants reported the least emotional bond with strangers (electronic supplementary material, figure S2). The strengths of the reported emotional bonds with friends were between those for primary and extended family members in both samples. A Mann–Whitney *U* test (after Holm–Bonferroni correction) on emotional bond yielded a significant difference only for the partner (*U* = 13 917, *p* ≈ 8 × 10^−9^, Holm–Bonferroni corrected), such that the emotional bond with the partner was lower in Japanese than in British participants.

The participants reported that being touched by their partner elicited most pleasantness, followed by their close relatives (electronic supplementary material, figure S3). Touch pleasantness and emotional bond were significantly correlated in both cultures, with Spearman correlation coefficient *r_s_*_4402_ = 0.69 in the British sample and *r_s_*_2977_ = 0.74 in the Japanese sample (*p*s < 10^−10^), The two cultures differed significantly in the degree of pleasantness of being touched by others; with the exception of sister and male stranger, the British reported finding social touch as more pleasant than did the Japanese (Mann–Whitney *U* test, *p*s in range [3 × 10^−16^, 0.015], Holm–Bonferroni corrected).

### (b) The relationship between emotional bond, pleasantness and touchability index

**Figure 2.**
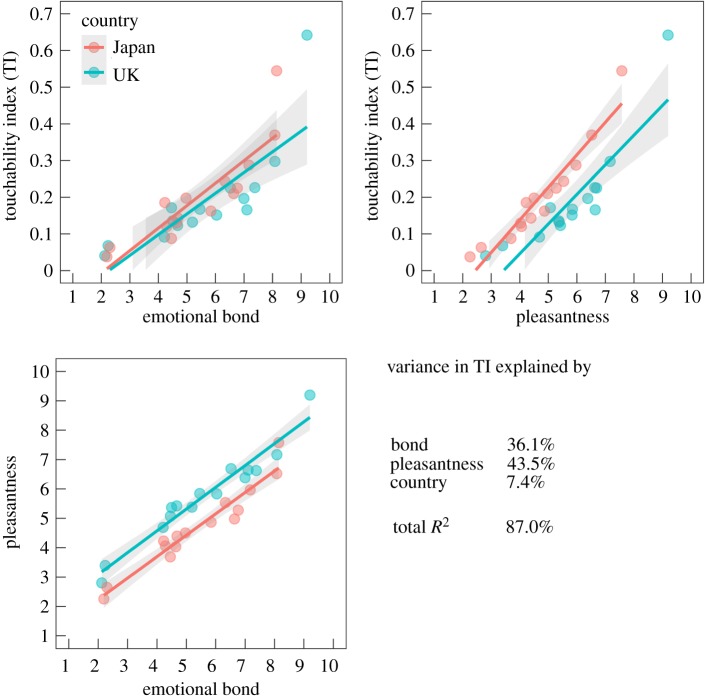
Correlations between touchability index (TI), emotional bond and pleasantness. Each dot represents the average response for one member of the social network in each culture (e.g. mother), with linear regression line and confidence interval for the regression fitted separately for each culture. Bottom right panel presents the relative importance of regressors in determining the TI in a linear model.

Figure 2 depicts the correlations between TI, pleasantness and emotional bond, with TI as the proportion of pixels in the body that a particular member of the participant's social network was allowed to touch (electronic supplementary material, figure S4). Linear multiple regression analysis with emotional bond, pleasantness of touch and culture as explanatory variables revealed that together these variables explained 20% of the variance in the total touchable body area (adjusted *R*^2^ = 0.20, *F*_3,7375_ = 626, *p* < 10^−10^). Bond (*β* = 0.18, *p* < 10^−10^), pleasantness (*β* = 0.31, *p* < 10^−10^) and culture (*β* = 0.16, *p* < 10^−10^) all predicted TI (see also electronic supplementary material, table S1 for a linear mixed effects model with subject as random effect). Partitioning *R*^2^ assigned 11.3% of the explanatory power to pleasantness of touch, 8.6% to emotional bond and only 0.4% of explanatory power to the cultural background (electronic supplementary material, figure S5). For the data averaged such that each social network member was represented by the average of responses about that person in each culture (figure 2), the adjusted *R*^2^ was 0.85, *F*_3,26_ = 58, *p* < 10^−10^.

Due to the high correlation between pleasantness and emotional bond, we also ran partial correlation tests. When controlling for pleasantness of the relationship, the association between emotional bond and TI was still significant, *r_s_*_7377_ = 0.15, *p* < 10^−10^, 95% CI [0.13, 0.17]. Similarly, when controlling for emotional bond, the association between pleasantness and TI remained significant, *r_s_*_7377_ = 0.22, *p* < 10^−10^, 95% CI [0.20, 0.24]. Thus, both pleasantness and emotional bond contributed independently to the relationship-specific TI.

Altogether, the analysis showed that emotional bond and perceived pleasantness of touch explained around 20% of the variance in TI, with only a negligible (0.4%) contribution from the culture. A supplementary analysis with emotional bond treated as categorical variable confirmed that emotional bond explains the variance in the total touchable body area (electronic supplementary material, figure S8). Finally, we confirmed this result by conducting the same regression analyses for both sexes in each culture (mean adjusted *R*^2^ = 0.21, range [0.17, 0.28]). Patterns of TI as a function of emotional bond were consistent regardless of the culture or toucher's sex (electronic supplementary material, figure S6).

### (c) Sex differences

**Figure 3.**
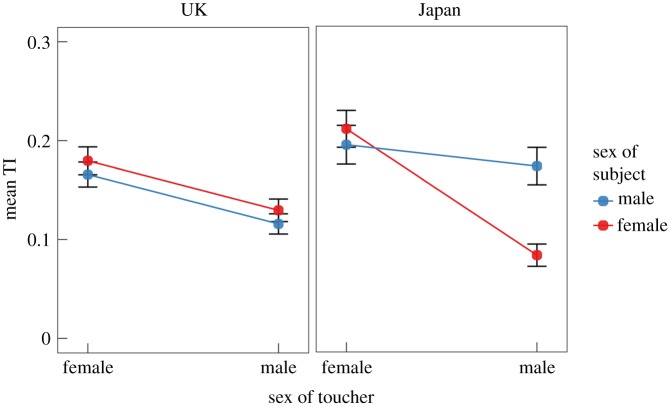
Interaction plot of the average TI for male and female participants (blue and red dots, respectively) with respect to male and female touchers for each culture. Error bars depict the 95% confidence interval. Red and blue lines indicate the interaction between toucher sex and the sex of the participant. Note: partners are excluded from the analyses, as the sex of the partner differs between the male and female participants.

We next examined if sex influences touch acceptance similarly in the UK and Japan. Figure 3 shows the relationship between touchable body area and sex of the toucher with respect to male and female participants (blue and red dots) in the UK and Japan. To statistically evaluate the effect of sex on TI, we conducted an ANOVA on the TIs of male and female participants and male and female touchers in both cultures. For partners, the sex of the partner was determined by whether the participant was male or female, and hence it was difficult to compare the effect of sex on TI between them. For this reason, we excluded the partner data from this analysis.

Three-way ANOVA (2 levels of cultures × 2 levels of toucher sex × 2 levels of participant's sex) on the TI revealed a significant main effect of culture *F*_1,6945_ = 12.6, *p* ≈ 4 × 10^−5^, *η*^2^ = 0.002, such that the TIs in the Japanese sample (*M* = 0.17, s.d. = 0.24) were larger than the TIs in the British sample (*M* = 0.15, s.d. = 0.20, *t*_5316.8_ = 3.38, *p* = 0.0007, 95% CI [0.008 0.029], *d* = 0.085). The main effect of the sex of the toucher was also significant *F*_1,6945_ = 137.9, *p* < 10^−10^, *η*^2^ = 0.019, such that the parameter estimates were larger for female touchers (*M* = 0.18, s.d. = 0.24) than for male touchers (*M* = 0.12, s.d. = 0.19, *t*_6739.3_ = 11.7, *p* < 10^−10^, 95% CI [0.050 0.071], *d* = 0.085). Thus, touch by female members of the social network was generally considered more acceptable across the whole social network (electronic supplementary material, figure S7). The effect of participant sex was not significant (*F*_1,6945_ = 0.72, *p* = 0.39).

All interactions were significant at the *p* < 0.05 significance level. Of the two-way interactions, the interaction between the toucher sex and participant sex was significant at *F*_1,6945_ = 18.5, *p* ≈ 2 × 10^−5^, *η*^2^ = 0.003, such that the difference between TI for female touchers and male touchers was larger for female participants (*M* = 0.08, s.d. = 0.11) than for male participants (*M* = 0.04, s.d. = 0.09, *t*_592.61_ = 5.7, *p* ≈ 2 × 10^−8^, *d* = 0.46). The interaction between the sex of the participant and the country was significant (*F*_1,6945_ = 22.6, *p* ≈ 2 × 10^−6^, *η*^2^ = 0.003). The TIs reported by Japanese male participants (*M* = 0.18, s.d. = 0.26) were higher on average than those reported by Japanese women (*M* = 0.15, s.d. = 0.23), British men (*M* = 0.14, s.d. = 0.20), or British women (*M* = 0.16, s.d. = 0.20), two sample *t*-test *t*-scores *t*_2712.5_ = 3.8, *p* = 0.0002, *d* = 0.14; *t*_2329.3_ *=* 5.3, *p* ≈ 10^−7^; and *t*_2496.4_ = 3.4, *p* = 0.0006, *d* = 0.13 respectively. The interaction between the toucher sex and country was significant (*F*_1,6945_ = 5.0, *p* = 0.025, *η*^2^ = 0.001). The TIs for Japanese female touchers (*M* = 0.20, s.d. = 0.26) were on average higher than those for British female touchers (*M* = 0.17, s.d. = 0.22) (*t*_2758.4_ = 3.7, *p* = 0.0003, *d* = 0.13). The three-way interaction was also significant (*F*_1,6945_ = 25.0, *p* ≈ 6 × 10^−7^, *η*^2^ = 0.003). This result seems to be mostly driven by the different responses of Japanese men and women with respect to male touchers (figure 3). Similar results were obtained using a mixed-effects model with subject as a random effect (electronic supplementary material, table S2).

### (d) Region-of-interest analysis

**Figure 4.**
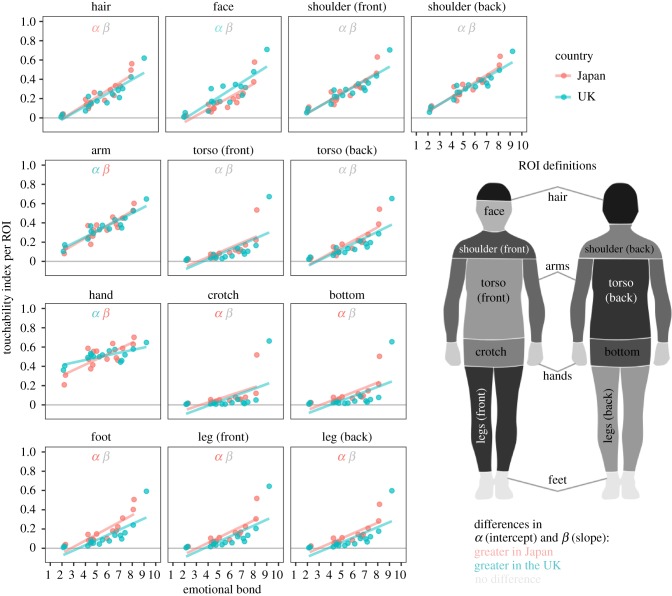
Visualization of ROI-specific cultural differences in TI versus emotional bond. Linear regression lines were fitted to each culture separately. Blue and red colours depict UK and Japan, respectively. Each dot represents the average response for one member of the social network (e.g. ‘British partner’ or ‘Japanese mother’). The grey intercept and slope terms mean no significant difference in the regression lines between the cultures. The coloured terms signal which culture had higher intercept (*α*) or slope (*β*) values, with blue and red colours indicating the British and the Japanese, respectively. The visualization is presented with averaged data in the interest of clarity, but the significance of intercept and slope are calculated from the full (un-averaged) data.

Whole-body TAM analyses revealed cultural differences in touchability of specific body areas, such as face, hand, and arm. To further examine for area-specific cultural differences, we next conducted linear regression analyses on the regional TIs for each culture, with emotional bond as the explanatory variable (figure 4). Adjusted *R*^2^ values of fitted linear functions averaged across body regions ranged between 0.04 and 0.15. Comparing linear models fitted to ROI-wise TI data showed significantly higher baseline acceptability (intercept) of touch on hair (*β* = −0.02, *t*_7376_ = −2.96, *p* = 0.003), feet (*β* = −0.05, *t*_7376_ = −7.3, *p* < 10^−10^), legs (both front, *β* = −0.03, *t*_7376_ = −5.3, *p* ≈ 9 × 10^−8^, and back, *β* = −0.03, *t*_7376_ = −5.7, *p* ≈ 2 × 10^−8^), crotch (*β* = −0.01, *t*_7376_ = −3.0, *p* = 0.003) and bottom (*β* = −0.03, *t*_7376_ = −5.1, *p* ≈ 3 × 10^−7^) in the Japanese sample. The same test showed higher baseline acceptability of touch on arms (*β* = 0.06, *t*_7375_ = 3.4, *p* = 0.0006), hands (*β* = 0.14, *t*_7375_ = 7.2, *p* < 10^−10^) and face (*β* = 0.07, *t*_7376_ = 9.3, *p* < 10^−10^) in the British sample. The same test showed a different rate of increase in TI for each unit of emotional bond (slope) on the arms (*β* = −0.01, *t*_7375_ = −3.5, *p* = 0.0004) and hands (*β* = −0.02, *t*_7375_ = −7.3, *p* < 10^−10^). In both of these ROIs, the TI was more responsive to changes in emotional bond (steeper slope) in the Japanese sample.

## 4. Discussion

The results show that although the touchable areas differed between the UK and Japan, they were similarly dependent on the strength of the emotional bond. Emotionally close individuals in the inner layers of the social network were allowed to touch larger bodily areas, whereas touching by strangers was primarily limited to the hands. The ROI analyses confirmed that most of the ROIs were similarly sensitive to emotional bond in both countries. This result suggests that the use of social touch for bonding purposes serves a similar function in East Asian and European cultures, rather than being merely culture-based normative behaviour. This interpretation accords with previous studies [9,19,23] suggesting cultural invariance in social touching.

We have previously reported consistent social touching patterns in a large sample of European cultures [9]. Additionally, American and Japanese college students were asked where they had touched and been touched by their parents, a same-sex friend and an opposite-sex friend [19]. The relative frequencies of touch by these different members of the social network followed the same pattern in both cultures [19].

One possible explanation for the cross-cultural similarities in social touching is the effect of globalization. For instance, Japanese culture may be influenced by social customs in e.g. Western movies that are shown widely in Japan. However, the relationship specificity of touchable areas (found here in European and Japanese cultures) is consistent with both our earlier study with Western cultures [9] as well as an older study looking at Japanese and North American cultures [19]. Thus, it is unlikely that the role of recently shared cultural elements plays a major role in the cross-culturally similar association between social touch and emotional bond.

Social touch induces positive feelings and improves interpersonal evaluation [24–27]. For example, participants evaluate even a stranger more positively, if that person has inconspicuously touched them during an interaction [25,27–29]. This close connection between social touching and preference toward the source of touch suggests a causal role of touch on social bonding. There is some evidence of touching causally impacting bonding in romantic relationships [30]. However, it is not clear if this extends to other relationships and this cannot be directly addressed from the current cross-sectional data. We also found that emotional bonds are correlated with experienced pleasantness of social touch. However, a cross-sectional design cannot reveal whether pleasure derived from being touched by someone enhances the emotional bond to the toucher, or whether liking someone makes their touch feel more pleasant.

Social touch is important for group cohesion of non-human primates, and grooming relationships predict the level of support during conflict situations and when in need of help [8,31–34]. It is hypothesized that grooming-induced relaxed and pleasurable feelings could constitute the psychological mechanism for an individual's willingness to offer subsequent help, thus forming the basis of mutual exchanges of social support [5,34]. Because social touching increases prosocial behaviour [35–37], it is possible that human social touch increases emotional bonding with the individual being touched, enhancing their prosocial behaviour.

Neurophysiological studies have revealed that the un-myelinated afferent c-tactile nerve fibres are selectively responsive to light and slow touch [38–40]. The signals carried out by these fibres are indirectly transmitted to the insula [38], a part of the neural network responsible for affective tactile interactions [40,41]. This pathway could thus support culturally universal hedonic nature of interpersonal touch. However, as shown in the present study, relationship information imposes top-down influences on this circuitry (e.g. how the individuals are touched and by whom they are touched, electronic supplementary material, figure S3). It is also known that physical contact with members of one's social network attenuates brain activity associated with both the threat of physical pain stimulus [42] and aversive images [43]. Processing interpersonal aspects of touch may recruit additional brain networks [44,45], including the action observation network (AON) [46]. Thus, the positive and calming effect of interpersonal tactile interchanges may be generated by the interaction of these brain networks, mediating the relationship between touchable area and emotional bonding, regardless of the different cultural norms.

### (a) Gender difference in both cultures

Participants in both cultures allowed women to touch their bodies more than men, which accords with prior findings in Western cultures [9,47]. The preference to touch by women was comparable in British men and women. By contrast, responses of the Japanese participants showed a clear gender difference: Japanese men did not show a statistically significant preference for touch by women, whereas Japanese women showed a remarkably strong preference.

Earlier studies have found opposite-sex touch to be preferable to same-sex touch [19,23,48–50]. Preference for female touch in the female participants might therefore seem to contradict earlier findings. On closer inspection, this seeming contradiction is caused by a difference in terminology. Most of the earlier studies discussing gender effects have inspected the difference of body accessibility for ‘same-sex friend’ and ‘opposite-sex friend’. Unfortunately, most of the older experiments [23,48–50] use the euphemism ‘opposite-sex friend’ to indicate a romantic partner (a usage made explicit in [51]), whereas ‘same-sex friend’ seems to refer to a platonic friend. This practice biases the comparison, as touching romantic partners is both qualitatively and quantitatively different from touching other people [9].

Taking this difference of nomenclature into account, our findings would in fact seem to be in line with earlier studies. For example, a similar preference for female touch has previously been found with respect to parents: participants touch and are touched more by their mothers than fathers, both in the United States [23,52] and in Japan [19]. Similar to the current findings, both male and female Japanese participants touch and are touched by their mothers more than by their fathers, but the difference is greater for female participants [19]. Conversely, in the American sample, the preference for mother over father was of comparable size between male and female participants [19].

### (b) Cultural differences between the Japanese and the British

While the overall association of touching and social bonding was concordant across cultures, some differences were also found. First, the Japanese participants reported the overall pleasantness of being touched to be lower than the British did (electronic supplementary material, figure S3), although both cultures showed similar changes in emotional bond across different touchers (figure 2). This difference may be in part due to differences in wording (‘kokochiyoi’ in Japanese versus pleasant in British), but it can also reflect differences in daily non-verbal communication: Japanese conduct social touching (e.g. handshakes and hugs) much less frequently than Americans [19] and they do likely less than British.

Second, we also observed three notable differences in the topographies of acceptable touch. The British participants allowed their partners to touch their bodies more than the Japanese (figure 1). This finding accords with previous research showing that touch between partners is more frequent in most body areas in the Western (North American) culture than in Japan [19]. The British participants in the present study also reported stronger emotional bonds with their partners than did the Japanese participants, suggesting that the difference between touch allowances for the partner might not be merely a cultural norm in behaviour but indicative of a wider difference in the intimate relationships in the two cultures.

Moreover, British participants allowed female family members and female friends to touch their faces more than did the Japanese participants. A previous study demonstrated that the British tend to touch their own faces more frequently than the Japanese do [53]. It is possible that such ‘accessibility’ to face may originate from gestures that are more specific to British than Japanese and their self-touching behaviour. By contrast, more Japanese participants reported that their female relatives are allowed to touch them on their legs and bottoms. Female members in the Japanese culture often take care of children in their network and have physical contact with them. It is considered that British culture is more individualistic than Japan and that individuals in such culture assume responsibility only for themselves and their immediate family [54]. Thus, the observed difference may be explained by the roles of the female relatives in child rearing in the two cultures.

We also observed cultural differences in the relationship between the emotional bond and the touchable area in specific body parts such as the arms and hands. In these regions, the strength of the emotional bond with a social network member increased touchability more in the Japanese versus British sample, i.e. the Japanese had a steeper slope in the fitted linear function (figure 4). This difference might be explained in terms of cultural difference in daily gestures. For instance, gestures involving physical contacts, such as handshakes and hugs, are more common in Western countries than in East Asian countries, such as Japan. This is supported by the higher intercepts in arm and hand for the UK sample in the ROI-wise analysis. Thus, more emotional closeness may be necessary for the Japanese to engage in this type of social touching.

Finally, the difference between the touch allowances for female touchers and male touchers was larger for Japanese women than for Japanese men or British participants of either sex (culture × sex of toucher × sex of participant interaction, figure 3). This culture-specific gender difference can be also associated with the above-mentioned points. More specifically, Japanese women had much smaller TIs for all males in their social network than for women with similar formal relationship (e.g. mother and father, see electronic supplementary material, figure S7). Collectively, although we found some differences, the associations between emotional bond, pleasantness and TI were concordant in both cultures.

### (c) Limitations

Our study was conducted online. Because the participants did not meet the experimenter, they could answer the questions without the sense of invasion of privacy (e.g. feeling embarrassed that experimenters in front of them know their touching behaviour). While there is some experimental evidence suggesting that attitudes towards touch impact touching behaviour [55], it is possible that the data may not directly translate to real-life touching behaviour. Therefore, it will be important to validate the current findings by conducting observational research of real-life touching in the future. Second, although the currently available evidence suggests that social touching is concordant across a wide range of Western, Orthodox and Japanese cultures (see also [9]), these data do not generalize to the other major cultures of the world [56]. However, already the present data demonstrate marked cross-cultural consistency in touch-dependent bonding across a wide range of cultures and geographical locations.

## 5. Conclusion

Relationship-specific emotional bonds account for the magnitude of social touching from social network members similarly between Western and East Asian (Japanese) cultures. Pleasure derived from touch, however, depended on the culture. Because the relation-specific social touching patterns of Japanese and Western respondents are consistent, the relationship between emotional bonding and touchable body area may be largely biologically determined. Human social touch, like social grooming of non-human primates, may provide a scaffold for social bonding with the members of one's social network. This supports the role of somatosensation and emotional feelings in the maintenance of social bonds in humans [57].

## Supplementary Material

Supplementary information

## Data accessibility

The data are available from the Dryad Digital Repository: https://doi.org/10.5061/dryad.f0f2642 [58] and the code to produce all the figures and numbers are available at https://version.aalto.fi/gitlab/jtsuvile/cultural-similarity-touch.
