# Supplementary material for: Cross-cultural similarity in relationship-specific social touching
Source: Proc Biol Sci. 2019 Apr 24;286(1901):20190467. doi: 10.1098/rspb.2019.0467 (PMC6501924; doi:10.1098/rspb.2019.0467)
Supplement: Supplementary information [file rspb20190467supp1.pdf]

Supplementary Information

Cross-cultural similarity in relationship-specific social touching

Juulia T. Suvilehto, Lauri Nummenmaa*, Tokiko Harada, Robin I. M. Dunbar, Riitta Hari, Robert Turner, Norihiro Sadato, Ryo Kitada*

***Corresponding author:**

Ryo Kitada, Division of Psychology, School of Social Sciences, Nanyang Technological University, 14 Nanyang Avenue, 637332, Singapore, Tel: +65-6316-8935; E-mail: ryokitada@ntu.edu.sg

Lauri Nummenmaa, Department of Psychology, University of Turku, Finland

Article DOI: 10.1098/rspb.2019.0467

Table S1

Mixed effects model for relation between TI, bond, and pleasantness. The model used was otherwise the same as within the main manuscript, but random effect of subject was added. Reference level for country was the UK.

Fixed effects				Random effects	
effect	estimate	Std error	t-value		Variance
Reference	-0.962561	0.043980	-21.886	Subject ID	0.3934
Emotional Bond	0.069898	0.004545	15.379	residual	0.4054
Pleasantness	0.136071	0.005226	26.038	# observations	7379
Country	-0.175810	0.053177	-3.306	# groups (subjects)	641

Wald chi-squared test for the linear mixed effects model

	Chi Sq	Df	p
Emotional Bond	237	1	$< 10^{-10}$
Pleasantness	678	1	$< 10^{-10}$
Country	11	1	0.0009

Table S2

Mixed effects model for gender effects. The model used was otherwise same as within the main manuscript, but random effect of subject was added. Reference levels for the fixed effects are: sex of participant: female; sex of toucher: female; country: the UK.

Fixed effects				Random effects	
effect	estimate	Std error	t-value		Variance
Reference	0.176	0.012731	13.823	Subject ID	0.02382
Sex of participant (SP)	-0.015	0.017137	-0.900	residual	0.02231
Sex of toucher (ST)	-0.052	0.006849	-7.587		
Country	0.032	0.019316	1.645		
SP * ST	0.0066	0.009303	0.704		
SP * Country	-0.0051	0.027026	-0.188		
ST * Country	-0.078	0.010367	-7.518	# observations	6953
SP * ST * Country	0.101	0.014578	6.940	# groups (subjects)	641

Wald chi-squared test for the linear mixed effects model

	Chi Sq	Df	p
Sex of Participant	0.19	1	0.66
Sex of Toucher	286	1	$< 10^{-10}$
Country	1.7	1	0.19
SP * ST	44	1	$< 10^{-10}$
SP * Country	2.9	1	0.09
ST * Country	13	1	0.0002
SP*ST*Country	48	1	$< 10^{-10}$

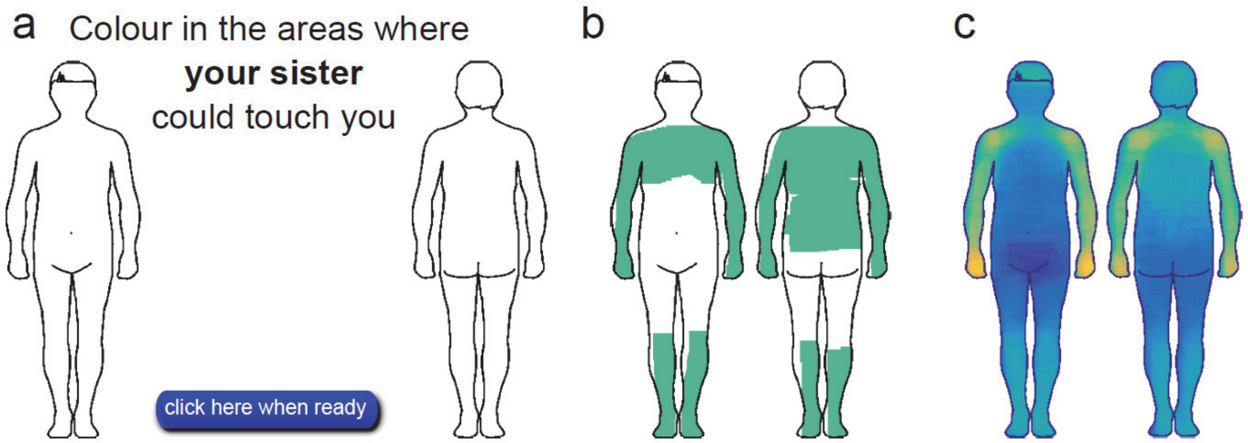

Figure S1: Participants were shown an initial screen with blank bodies and instructed to color bodily areas where the specified social network member would be allowed to touch them (a). Resulting participant-wise Touch Space Maps (b) were evaluated using random effects group analysis (c) to reveal relationship-specific patterns of social touch in East Asian (Japanese) and Western (British) cultures.

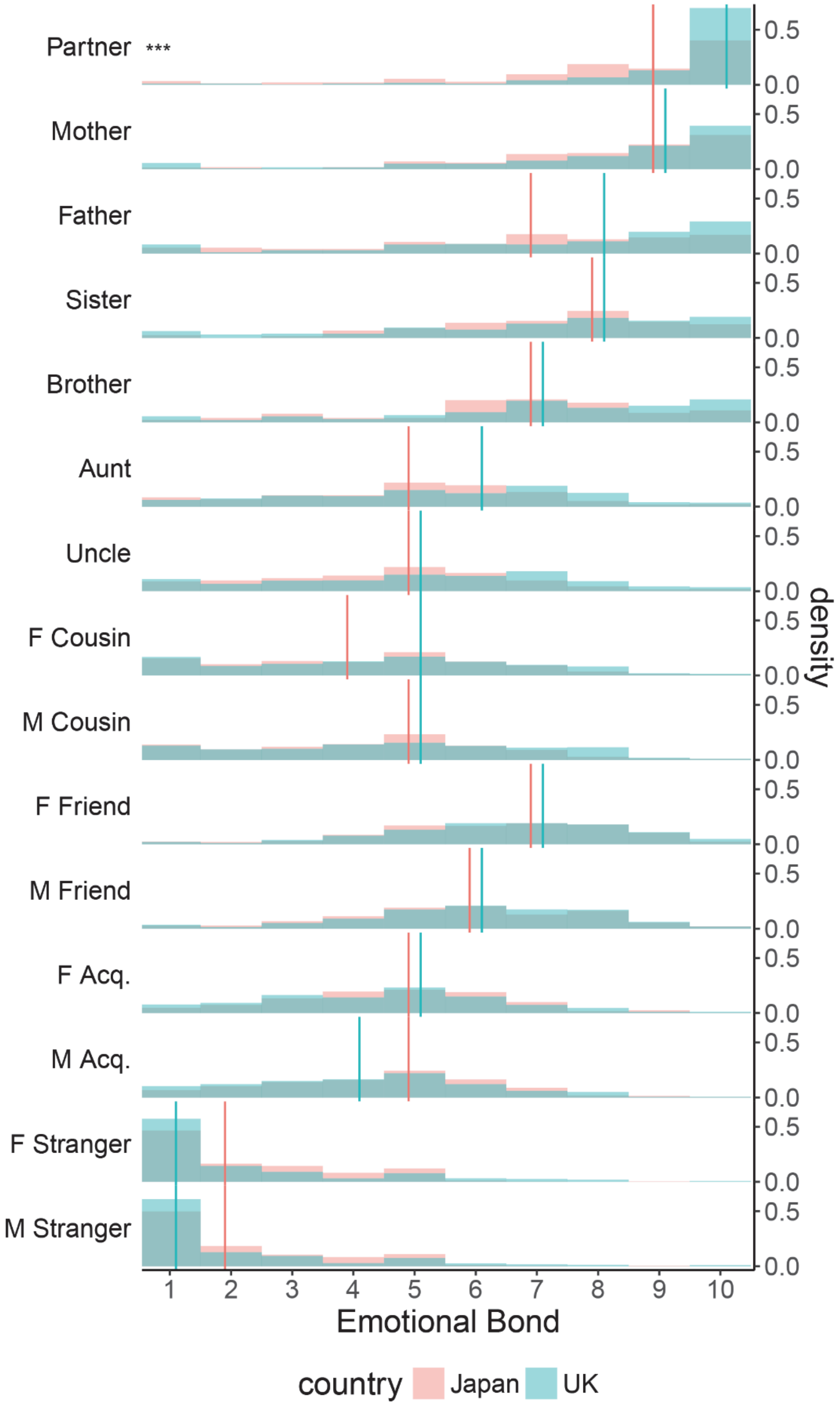

Figure S2: Distribution of emotional bond for each social network member in Japanese (red) and British (turquoise) participants. Vertical bars indicate means, asterisks statistically significant cultural differences in Mann-Whitney U test, with *** denoting $p<0.001$. F and M indicate female and male members, respectively.

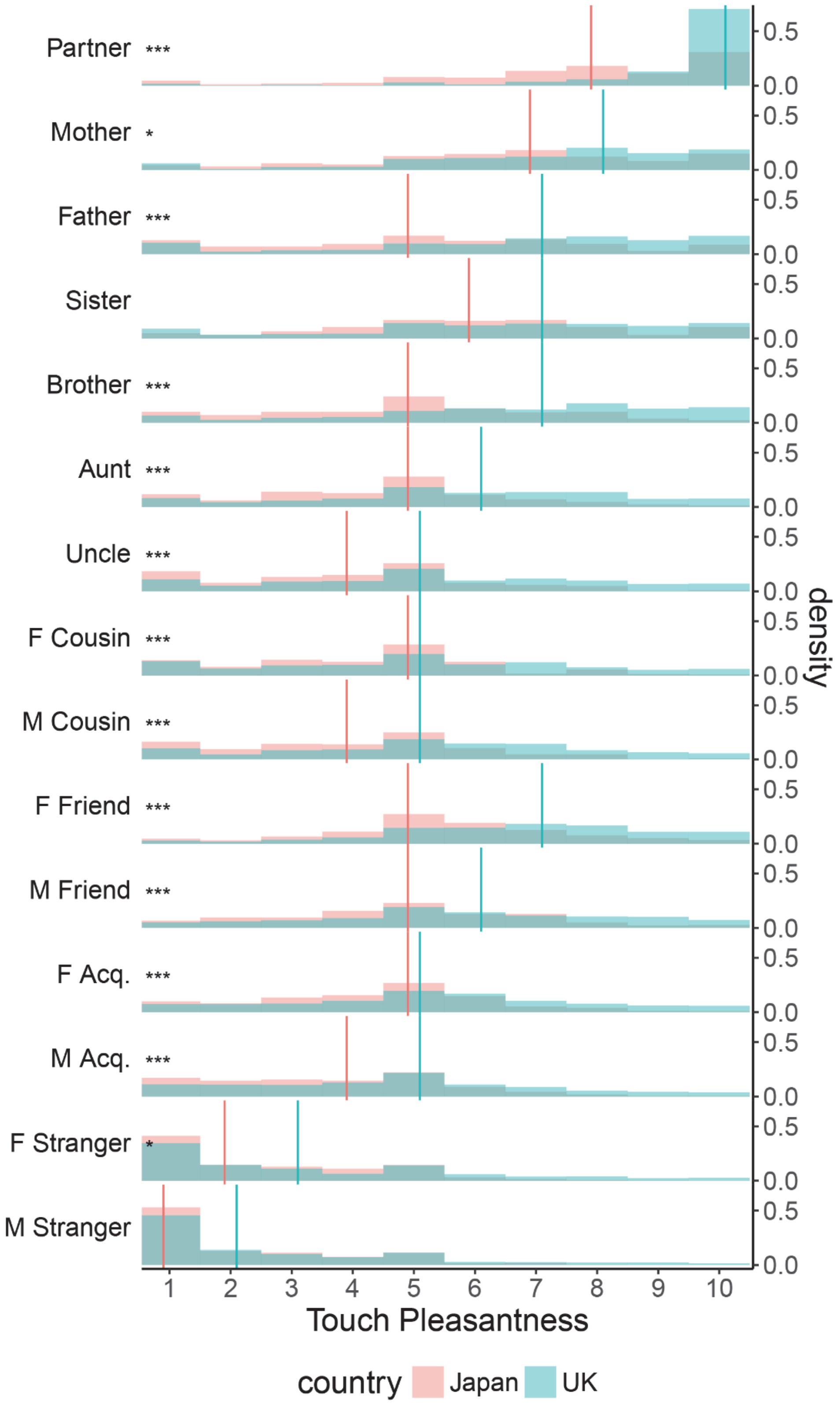

Figure S3 Distribution of perceived pleasantness of touch. Asterisks indicate the results of Mann-Whitney U tests, with * $p < 0.05$, ** $p < 0.01$, *** $p < 0.001$, Holm-Bonferroni corrected. Almost all of the means are significantly different such that British participants find touch consistently more pleasant than the Japanese participants regardless of social relationship. F and M indicate female and male members, respectively.

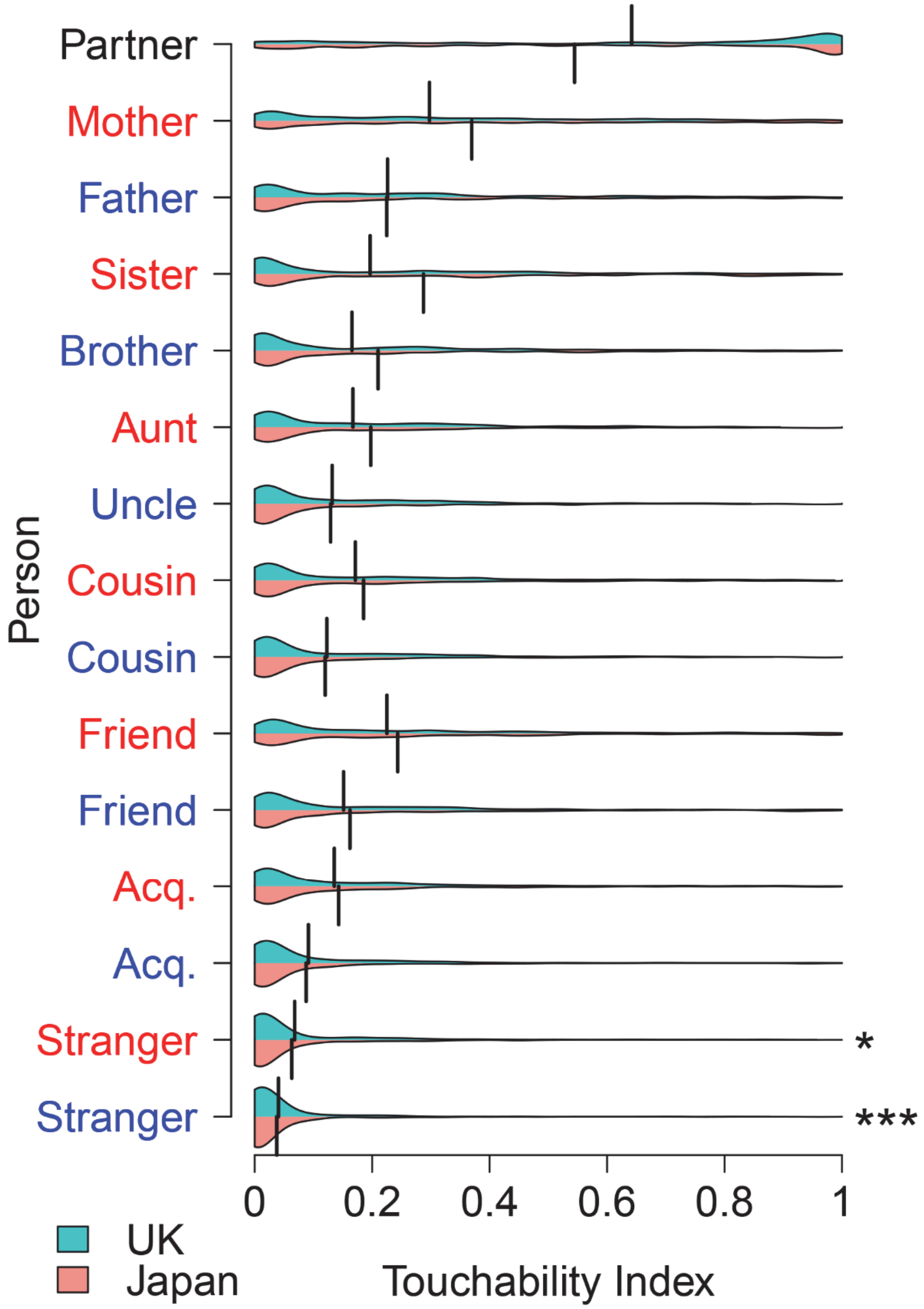

Figure S4 Distribution of Touchability Index for each member of social network. The members of the social network whose TI is significantly different in British and Japanese samples, according to a two-sample Kolmgorov-Smirnov test, are female stranger (D = 0.15, p = 0.02) and male stranger (D = 0.23, p < 0.0001). Reported p-values Holm-Bonferroni corrected. Red and blue labels indicate female and male members of the social network, respectively.

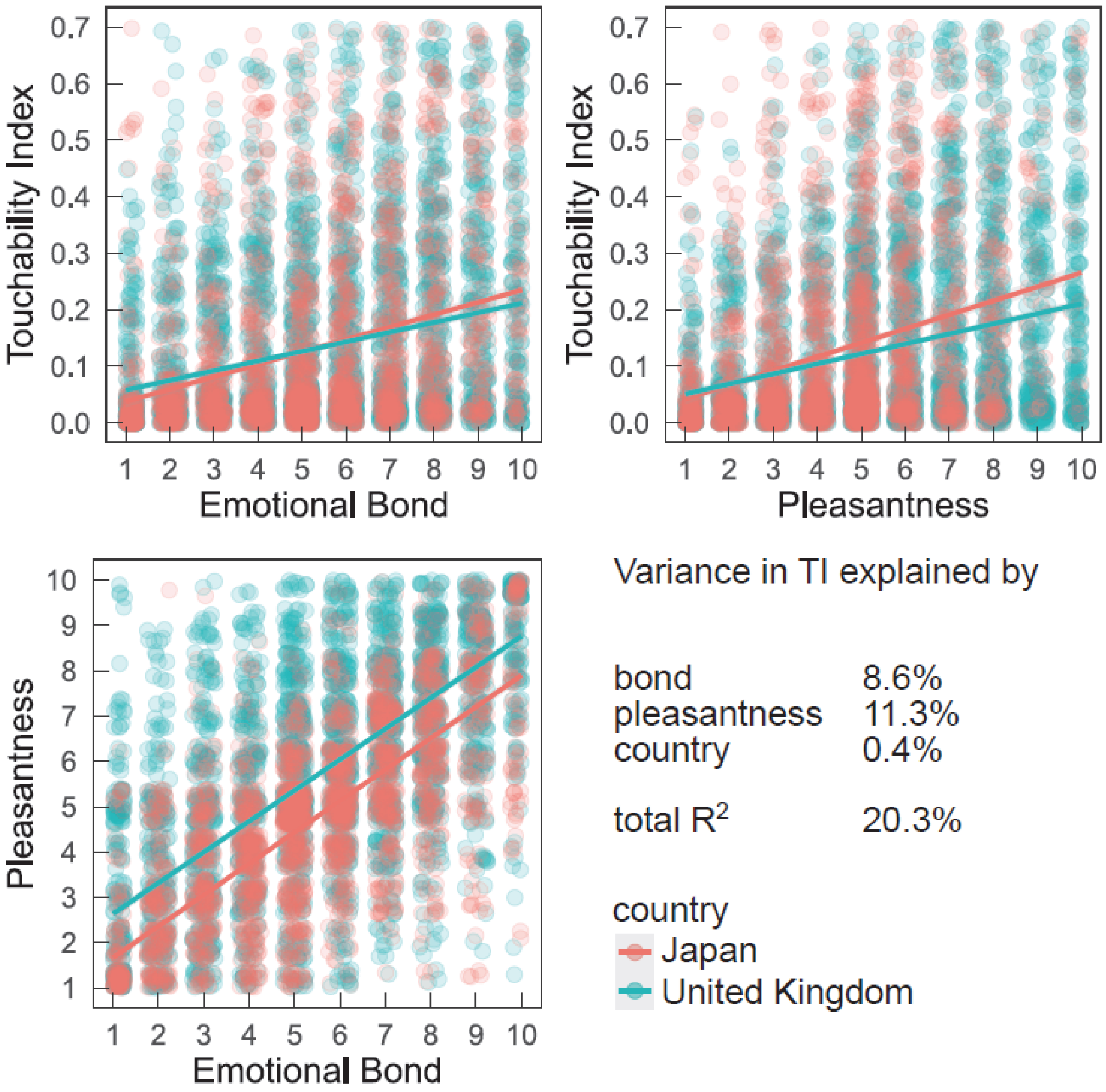

Figure S5 Correlations between TI, emotional bond, and pleasantness for un-averaged data. Each dot represents the response of one participant for one member in their social network. Visual jitter was added to make responses to discrete variables (emotional bond and pleasantness) distinguishable. Linear regression line and confidence interval for the regression are fitted separately for each culture. Bottom right panel presents relative importance of regressors in determining the TI in a linear model for this un-averaged data.

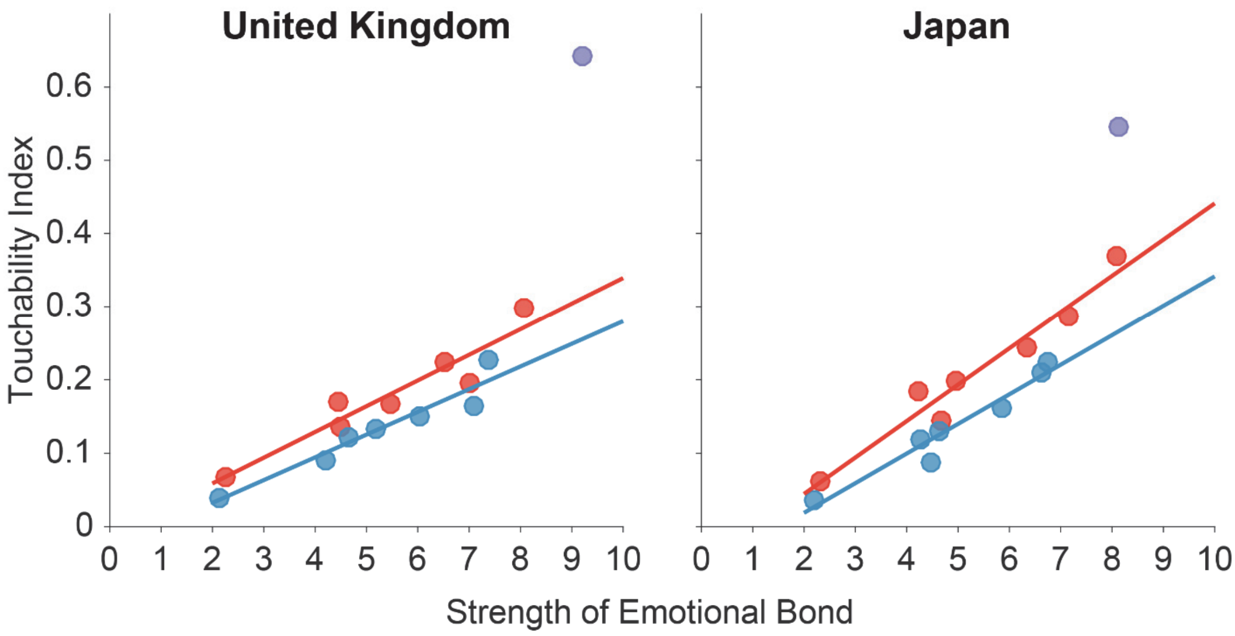

Figure S6 Sex differences in the relationship between emotional bond and touchability index. Each dot represents average response for one member of the social network. Red and blue lines indicate the fitted regression line to female and male members of the social network, respectively. The sex of the participant does not impact the slope of the regression. This analysis excludes the partner, as the sex of the partner differs between the participants, and therefore the data point denoting partner is shown as purple.

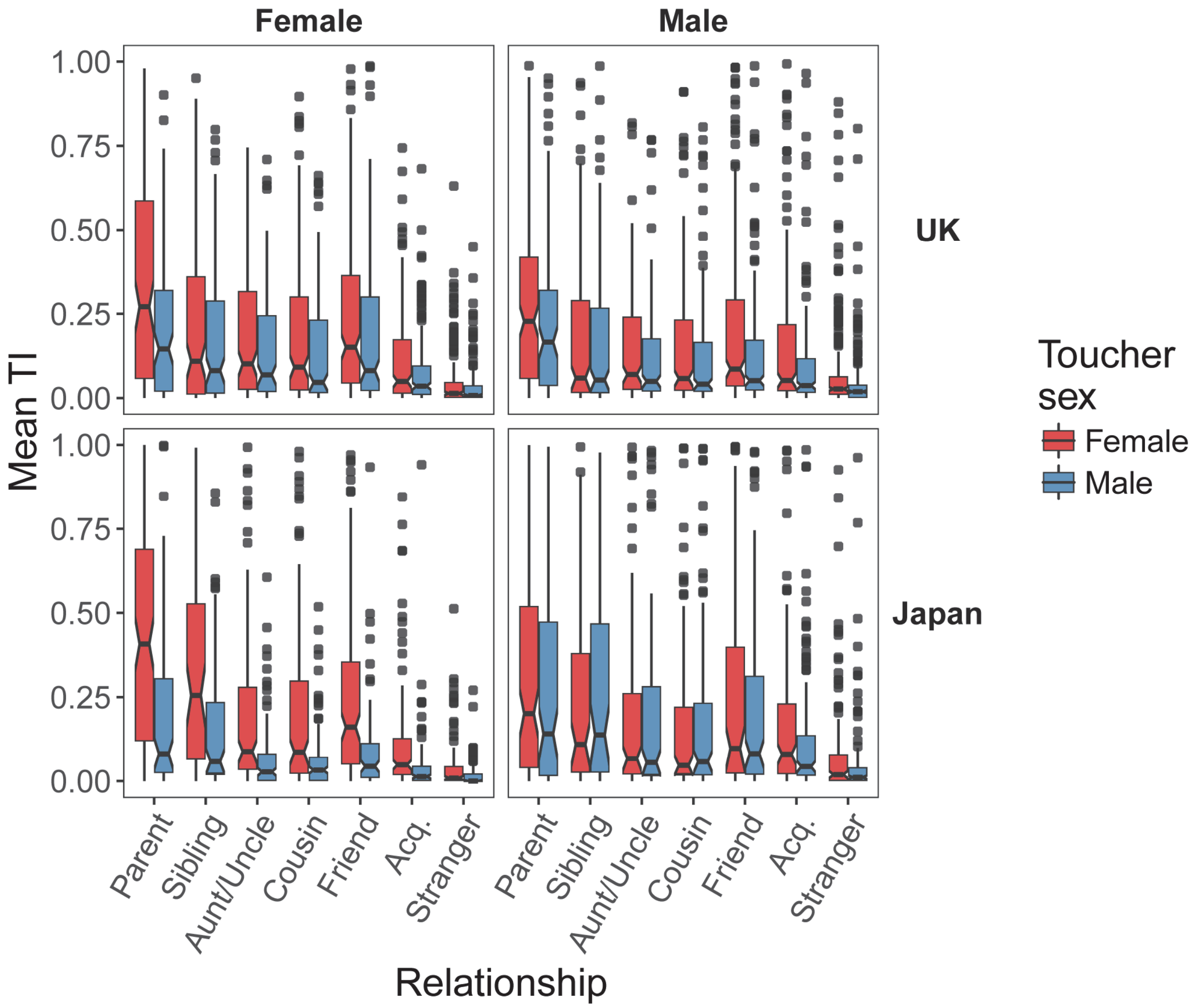

Figure S7 Mean touchability indices for different social network members in British (top row) and Japanese (bottom row) cultures. Data shown separately for female and male participants (left and right columns, respectively). X-axis shows the relationship of the toucher with the participant, while red and blue bars indicate female and male touchers respectively. Error bars show SEM.

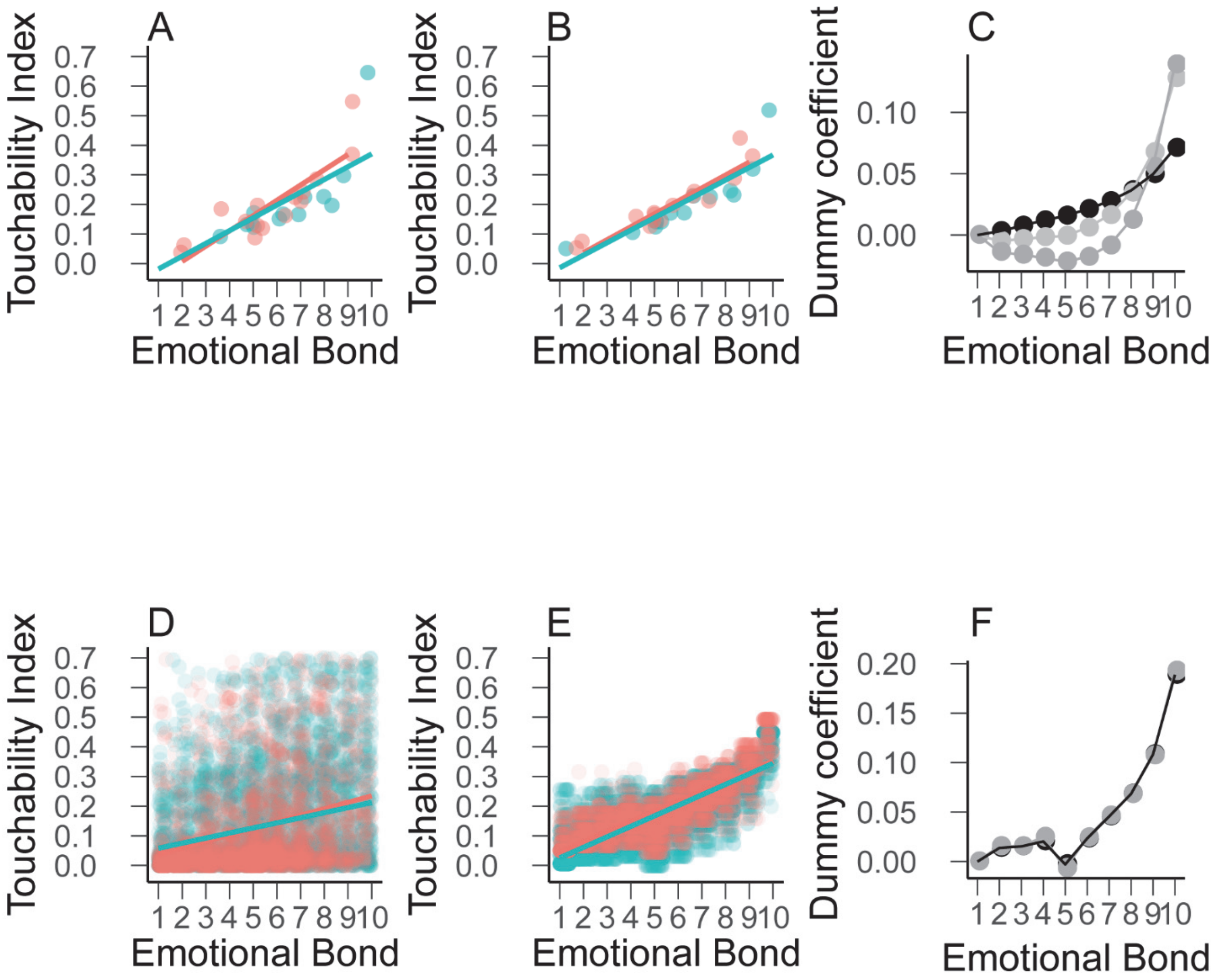

Figure S8 (A,D) TI as a function of the emotional bond scores in British and Japanese samples. (B,E) Fitted response variables as determined by penalizing sum of squared differences of adjacent dummy coefficients, using smoothing parameter lambda = 5. (C,F) Group lasso estimates of dummy coefficients as a function of levels of emotional bond for lambda = [5, 10, 50], darker colour denotes higher lambda. Data are presented both averaged over each social network member (A-C) and not averaged (D-F). Visual jitter has been added to A, B, D, and E for visualisation purposes.
